# What does the immunometabolic status tell us about depression?

**DOI:** 10.1192/j.eurpsy.2023.46

**Published:** 2023-07-19

**Authors:** P. Lopez-Garcia

**Affiliations:** Psychiatry, Universidad Autonoma de Madrid, Madrid, Spain

## Abstract

**Abstract:**

Despite being a clinical identifiable entity, major depressive disorder (MDD) is an heterogenous clinical syndrome, with a variety of clinical presentations which likely reflects different biological underpinnings. The identification of biologically-based depression symptoms profiles would be of great importance to unravel different pathophysiological pathways in MDD and therefore to achieve more precise and personalized therapeutical approaches as well as preventive strategies.

Converging evidence from epidemiological and clinical studies, points to the importance of inflammation in MDD, shown by increased levels of pro-inflammatory proteins and increased inflammation-related comorbidities, including metabolic diseases. In fact, there exists a bidirectional relationship between inflammation and metabolic dysfunction that could be linked to multiple factors, including life style, stress and genetic predisposition. MDD patients exhibit several metabolic disturbances such as overweight, insuline resistance and dyslipidemia, among others, which are not always fully explained by life style factors. These findings have led to the formulation of an immunometabolic hypothesis, which could be present in a subgroup of MDD patients, associated to specific symptoms and clinical features.

In this presentation, data reflecting the complex relationships and interactions between immune and metabolic disturbances in MDD will be shown. In particular, it will be shown how machine learning approaches can be useful to disentangle the clinical and biological heterogeneity of MDD, using immunometabolic biomarkers.

**Disclosure of Interest:**

P. Lopez-Garcia Grant / Research support from: Grant from Carlos III Health Institute ref PI15/00204 and FEDER funding from the EU

